# Distance comparisons in virtual reality: effects of path, context, and age

**DOI:** 10.3389/fpsyg.2015.01103

**Published:** 2015-08-14

**Authors:** Ineke J. M. van der Ham, Heleen Baalbergen, Peter G. M. van der Heijden, Albert Postma, Merel Braspenning, Milan N. A. van der Kuil

**Affiliations:** ^1^Health, Medical, and Neuropsychology, Leiden University, Leiden, Netherlands; ^2^Experimental Psychology, Utrecht University, Utrecht, Netherlands; ^3^Methodology and Statistics, Social Science, Utrecht University, Utrecht, Netherlands; ^4^Southampton Statistical Science Research Institute, University of Southampton, Southampton, UK; ^5^Department of Neurology, Utrecht University Medical Center, Utrecht, Netherlands

**Keywords:** distance comparison, path, context, virtual reality, age, gender

## Abstract

In this large scale, individual differences study (N = 521), the effects of cardinal axes of an environment and the path taken between locations on distance comparisons were assessed. The main goal was to identify if and to what extent previous findings in simple 2D tasks can be generalized to a more dynamic, three-dimensional virtual reality environment. Moreover, effects of age and gender were assessed. After memorizing the locations of six objects in a circular environment, participants were asked to judge the distance between objects they encountered. Results indicate that categorization (based on the cardinal axes) was present, as distances within one quadrant were judged as being closer together, even when no visual indication of the cardinal axes was given. Moreover, strong effects of the path taken between object locations were found; objects that were near on the path taken were perceived as being closer together than objects that were further apart on this path, regardless of the metric distance between the objects. Males outperformed females in distance comparison, but did not differ in the extent of the categorization and path effects. Age also affected performance; the categorization and path effects were highly similar across the age range tested, but the general ability to estimate distances does show a clear pattern increase during development and decrease with aging.

## Introduction

Memory for locations is a key element of spatial cognition and is relevant at different spatial scales. We are often required to remember locations of objects in order to proceed with our daily activities, for instance when we need to locate our keys. At a much larger spatial scale similar abilities are required, like remembering where the bus stop is, to be able to arrive at our destination. Typically, humans are not very accurate in memorizing the precise metric properties of locations. A hierarchical approach has been applied to characterize this process, separating categorical location processing from a fine-grained location encoding (Huttenlocher et al., 1991). Especially when our memory is tested with longer delays, biases tend to emerge that lead to systematic deviations from the actual location. This has been assessed with simple stimuli, like remembering a dot location within a circle (e.g., Postma et al., 2006). Over time, participants tend to rely more on categorization; a larger deviation from the actual locations toward the diagonal axes of the circle, which form the stereotypical locations of the four quadrants comprising the circle, with the cardinal axes as the boundaries of these quadrants. Similar findings of categorization have been reported for other stimulus designs (e.g., Werner and Diedrichsen, 2002; see, however, Plumert and Hund, 2001).

At a larger spatial scale categorization effects have also been observed. Recall of landmarks from a natural, campus environment shows clear influences of non-spatial, hierarchical information factors (Hirtle and Jonides, 1985). Importantly, in addition to these findings of categorization, it has been observed that at this larger scale, when navigating, participants also show systematic distortions in their estimations and representations of remembered locations (e.g., Stevens and Coupe, 1978; Foo et al., 2005). This can be considered a so-called path effect; the path a person takes in an environment distorts the metrics of the spatial representation of the environment. For instance, straight parts of a route appear to be shorter than they are, in particular when no relevant information is provided when moving along that part of the route (see e.g., Sadalla and Magel, 1980; Passini, 1984; Giraudo and Pailhous, 1994; Hochmair and Frank, 2000). Similar effects have been found for virtual environments (e.g., Cubukcu and Nasar, 2005). Moreover, even methods to reduce such effects have been reported, such as “minification” in which a larger field of view is presented to reduce underestimation of egocentric distances (e.g., Kuhl et al., 2006; Zhang et al., 2012).

Whereas our spatial memories of the outside world clearly can be affected by perceptual categorization biases as well as by movement-related path effects, the two have rarely been investigated together. In the current study we employed a controlled dynamic virtual reality (VR) task and focused on whether such categorization and path effects exist in three-dimensional VR environments. Moreover, we examined the interaction between categorization and path effects, to further understanding of these fundamental processes in spatial memory. We used circular virtual 3D environments. This allowed us to create an environment in which no contextual cues to spatial locations were available, so locations had to be encoded based on their metric properties. Moreover, this enabled us to study the categorization effect based on cardinal axes as has been found for the 2D circular stimulus, by creating artificial quadrant boundaries by means of color cues on the wall. A comparison of performance in an environment with and without categories could then reveal to what extent spatial representations in 3D are affected by overt categorization. To this aim, the main task in the current experiment was to compare the distances between objects encountered in the environment. In these distance comparisons (see e.g., Denis and Zimmer, 1992; Noordzij and Postma, 2005), participants memorize locations and are asked questions like; “At location A, which one is closer, location B or location C?” Locations B and C were chosen to reflect different combinations of locations within and outside the category of location A. Given the categorization effect in 2D, we expected to find similar categorization in 3D; locations within the same category are be perceived as being nearer to each other than locations in two different categories.

The current experimental set-up was dynamic in nature, as participants moved from one object to the next when they memorized object locations. This spatiotemporal layout of the objects in the environment allowed the assessment of potential path effects in addition to the quality of location memory in itself. We expected to find such a path congruency effect in our study; when two locations are positioned closer together along the path traveled during memorization, participants will perceive them to be metrically closer to each other.

An important influential factor to consider when studying these aspects of spatial cognition concerns individual differences. Individuals tend to vary substantially in their spatial skills and strategies (e.g., Hegarty and Waller, 2005; Hegarty et al., 2006). In particular, differences in age and gender can have a considerable impact on spatial memory. Most location memory studies have been performed in young, healthy adults. Yet, the categorical bias in 2D studies has also been observed in young children and has inspired developmental theory on spatial cognition (Huttenlocher et al., 1994). In this influential study it has been shown that at an age of 16 months, children are able to use distance and category information to estimate location. Yet, more adult-like categorization influences appear from age 9 (Sandberg et al., 1996), with early signs of some categorization already present in 4-year olds (Huttenlocher et al., 1994). In contrast, others report that children are affected by category in their distance estimates from age 11 onwards, and that the adult pattern of performance is achieved even later in development (Plumert and Hund, 2001). It is therefore unclear what the precise developmental pattern is for distance comparison. Young children appear to be able to use categorical information from an environment, but may do so differently than young adults, depending on the task at hand.

In addition to studying the developmental pattern of performance, we also examined aging. In general, aging has also shown to affect spatial cognition in distinct ways (for a review, see Klencklen et al., 2012). For instance, Cherry and Park (1993) have shown that as general working memory capacity decreases, this could lead to impairment in location memory as well. Age-related decrease in spatial memory has been found for tasks in virtual environments (e.g., Moffat et al., 2001; Head and Isom, 2010) and is linked to reduced neuroanatomical volumes, particularly of the hippocampus (e.g., Moffat et al., 2007; Wiener et al., 2013). As older adults are more prone to use an egocentric, or observer based, strategy, as opposed to an allocentric, environment based, strategy during navigation (Rodgers et al., 2012), it could well be that with older age, less attention is paid to the environment and its potential cues. This would suggest a weaker effect of categorization with increased age. In contrast, a stronger egocentric strategy could also boost the path effect, as more attention is paid to the path traveled. In light of these considerations it is of importance to gain further insight in the developmental and aging patterns of distance comparisons in a virtual 3D environment. Therefore, in the current study a very large sample of participants was included with ages ranging from 5 to 78 years old. With this wide age span we created the unique opportunity to examine distance comparison performance across the lifespan within a single study.

As a secondary factor in individual differences, gender was also considered. Gender has also been shown to be a powerful factor in explaining individual variation in location memory performance. A general finding concerning gender is that males tend to outperform females when basic (geo)metric knowledge is required (e.g., Castelli et al., 2008; Holden et al., 2015). This difference typically disappears when more contextual information is available, such as clear landmarks and route information (e.g., Lawton, 1994; Kober and Neuper, 2011). In the current task design this could mean that females may show stronger effects of categorization (see, also Holden et al., 2015) and are more strongly affected by the path taken. When also taking into account age for a large sample of participants, as we did in the current study, it is possible to further track the potential effect of gender across the lifespan.

Taken together, in the present study we examined distance comparisons in a three dimensional virtual environment in a large and varied sample of participants. The impact of visible spatial categories in the environment was assessed, as was the impact of path memory. Both gender and age were taken into account as between-subjects variables.

## Materials and Methods

### Participants

In total there were 521 participants in the experiment; 278 children (5–17 years old), 107 younger adults (18–40 years old), and 136 older adults (41–78 years old). In Table 1 demographic details for these three groups are provided. The experiment was part of “Science Live,” an initiative of Nemo Science Center, Amsterdam, for which ethical approval was obtained prior to the study. All participants were visitors of this science museum, who were asked to participate during their visit. All participants, or their parents when underage, were required to sign an informed consent form prior to participation.

**TABLE 1:** **Descriptives of the three participant groups**.

****	****	**Children**	**Younger adults**	**Older adults**
N		278	107	136
% male		59.0	54.0	52.0
Age (in years)	Mean	9.9 (2.3)	30.2 (7.2)	49.5 (8.7)
	Range	5–17	18–40	41–78

Standard deviations in parentheses.

### Materials and Task Design

The Blender open source 3D package (The Blender Foundation Amsterdam, Netherlands; www.blender.org; Version 2.70) was used to create the three dimensional environment in which the experiment was set. Tasks were administered in Blender’s Game Engine module. The tasks were presented on a desktop monitor and auditory samples were played through headphones (Sennheiser 201 HD). Note that participants were seated throughout the experiment, and controlled movement through the virtual environment with button presses.

Regardless of age, participants took part in the same experiment. In order to facilitate the younger participants in particular, a narrative structure was applied. A short background story was introduced, in which the participants were asked to help a robot gather all objects on different floors of a space ship. The robot served as a medium to provide instructions and training for all tasks. All instructions were provided orally through headphones, so reading proficiency could not affect the level of understanding of the instructions.

Participants navigated through the environment from a first-person perspective. The up and down keys of a keyboard were used for translational motion. The left and right buttons were used for rotation. The space key was used to pick up objects and the enter key was used to drop objects. The experiment started with a practice run in order to get participants accustomed to the interaction with the environment. Movement was practiced by following the robot through a series of corridors with several turns. Interaction with objects was practiced by having participants pick up and drop an object on a specific red circle on the floor. Completion of all aspects of the practice run was required to continue to the actual experiment.

Participants completed a distance comparison and a location estimation task. Both tasks consisted of two conditions which were tested in separate trials. As such, each condition corresponded to one of four floors in the spaceship. These conditions were presented in semi-randomized order (always grouping the trials of a task together), in order to avoid systematic errors. Note that in the current paper, we focus on the distance comparison task. Since the location estimation task consisted of placing back individually presented objects, it did not concern the examination of categorization and path effects. Therefore, location estimation performance is not discussed in the current paper.

**FIGURE 1:**
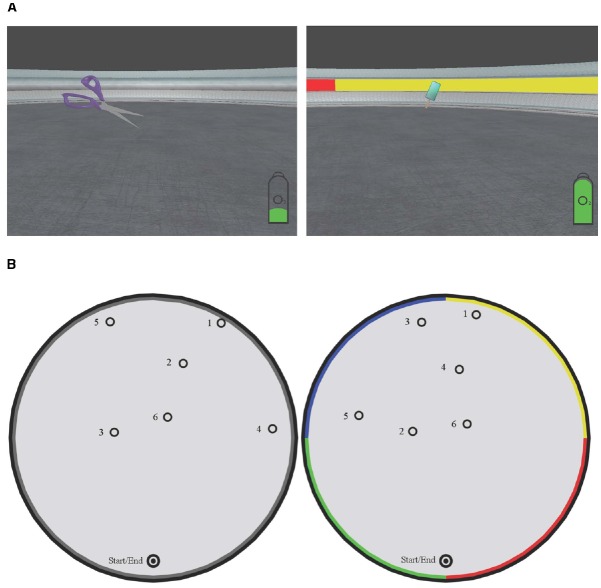
**(A)** Top view of the two different conditions; the color cue condition and the no color cue condition. In both images, the positions of the six sequential objects are indicated. Two different, but highly comparable layouts of objects were used for each participant, evenly divided between the two conditions. **(B)** Screenshots taken in the environment, from the participant’s perspective.

The conditions in the distance estimation task were *color* and *non-color*. In the color condition, the trial took place in a circular environment in which a horizontal colored line was shown along the wall. The wall was divided into four equal segments with blue, red, green and yellow textures, as shown in Figure 1. In the non-color condition, the same circular environment was used with the colors on the wall replaced by a single gray texture. In Figure 1, both conditions are depicted from the participant’s perspective and from above, with one of the object layouts used as an example. Participants received similar instructions in the color and non-color conditions.

Each condition consisted of the sequential presentation of six objects. The participant was asked to pick up these six objects sequentially. When the first object was picked up, by moving toward it and pressing the appropriate button, the second object would appear, and so forth. The participants were instructed to travel along straight lines directly toward objects as much as possible. The length of the traveled paths was recorded to exclude trials in which participants deviated too much from these straight lines. In this way, all participants traveled along the same paths from object to object. When the last object was picked up, participants were asked to move back to an elevator. In the elevator participants were tested on their knowledge of the object’s positions.

For the *distance comparison* questions, participants were presented with one of the objects, the “target,” at the top of the screen and two of the objects at the bottom of the screen. Objects were presented on a neutral background, without any cues to where they were presented during the study phase. The question was which one of the two bottom objects was closest to the object at the top. Six trials were presented. Those trials were composed based on the locations of the objects with regard to the category boundaries (the four edges of the colored quadrants on the wall) and the path traveled between the six objects. The correct answer could either be within the same category as the target or in a different category. Note that in each trial these category characteristics were used, regardless of whether color cues were present or not. Cardinal axes used to determine category boundaries (either overt—color, or cover—no color) were based on the participants’ starting position. Also, the metrically nearest object could be nearest or farthest in the path taken, so it was either congruent or incongruent with the path taken. Six different distance comparison conditions were created, as described in Table 2. For each trial a different configuration of the six objects was used. These configurations were composed to accommodate the different conditions. Performance was measured by calculating percentage correct for each condition, with 50% as chance level for all trials.

**TABLE 2:** **A description of the six conditions used in the distance comparison task**.

**Condition**	**Category**	**Path**
1	Neutral	Congruent
2	Neutral	Incongruent
3	Congruent	Congruent
4	Incongruent	Congruent
5	Congruent	Incongruent
6	Incongruent	Incongruent

Three objects were presented for each trial. The top object was the cue, the other two objects presented simultaneously at the bottom of the screen, were the two possible answers to the question “Which object was closer?” Therefore the nearest object represents the correct answer and the farthest object the incorrect answer. The distance between the target and the farthest object was approximately twice as large as the distance between the target and the nearest object. Category indicates the colored segment of the floor the object was in. Path indicates whether the object would also be closest with regard to the path that was taken between the objects.

### Procedure

Participants were recruited in the museum or visited the testing area at their own initiative. First, they were informed about the nature of the experiment. If they agreed to participate, they signed an informed consent form, or a parent signed it, if the participant was under the age of 18. The participants were brought to a separate room of the museum, where they could perform the experiment in quiet, controlled conditions. Two participants could perform the experiment at the same time, on two identical computers, but each participant performed the experiment individually. At least two experimenters were present at all times to ensure that participants were fully focused on the experiment and could ask questions if necessary. All instructions were provided verbally through headphones so participants could complete the experiment without further assistance. After completion of the experiment, participants were debriefed about the goals of the experiment and expected outcome. They were specifically instructed not to discuss the experiment with or near other (potential) participants in the museum, to avoid participants who could be informed about the details of the experiment.

### Statistical Analyses

Multilevel logistic regression analysis (Hox, 2012, chapter 6) was used to investigate which factors influenced performance. Multilevel analysis (ML) was used because each participant had 12 responses as the within-subject design was Category (3) × Path (2) × Color (2). ML is an alternative to repeated measures ANOVA and MANOVA where each of the responses leads to a record, and the model takes into account the records of each participant may be dependent. One advantage of ML is that it can easily deal with missing values on the dependent variable accuracy as records where performance is missing, are ignored. A second advantage is that it is more flexible than (M)ANOVA in the sense that it can be used for dependent variables that are not quantitative, i.e., accuracy is dichotomous (correct/incorrect) and for dichotomous variables such as performance logistic regression is appropriate, hence we use ML logistic regression. In the current dataset there is a two-level structure, because there are 12 responses (level 1) within each participant (level 2), and there are explanatory variables at level 1 (Category, Path, and Color) and at level 2 (Age and Gender).

For the analysis strategy we follow recommendations of Hox (2012, chapter 4.1). We start with a so—called empty model (a model without explanatory variables) and a random intercept, which shows whether there are individual differences in the participants (some are more accurate than others). Then, as a second step, main effects and interaction effects of the variables at the first level are added to the model. In the third step the second level variables are investigated. And at the fourth step we investigate whether there are random effects for the variables found to be significant at the first step: this may show that a factor is on average significant but that there are individual differences in the size of the effect, that can possibly be explained by the variables at the second level (a so-called cross-level interaction). The above shows the significance of effects. For relevance we calculate so-called partial effects, i.e., the average difference in probability in performance (Long, 1997).

The model is estimated using HLM (Raudenbush et al., 2004) using maximum likelihood; hence for model comparisons likelihood ratio difference tests are used.

## Results

**FIGURE 2:**
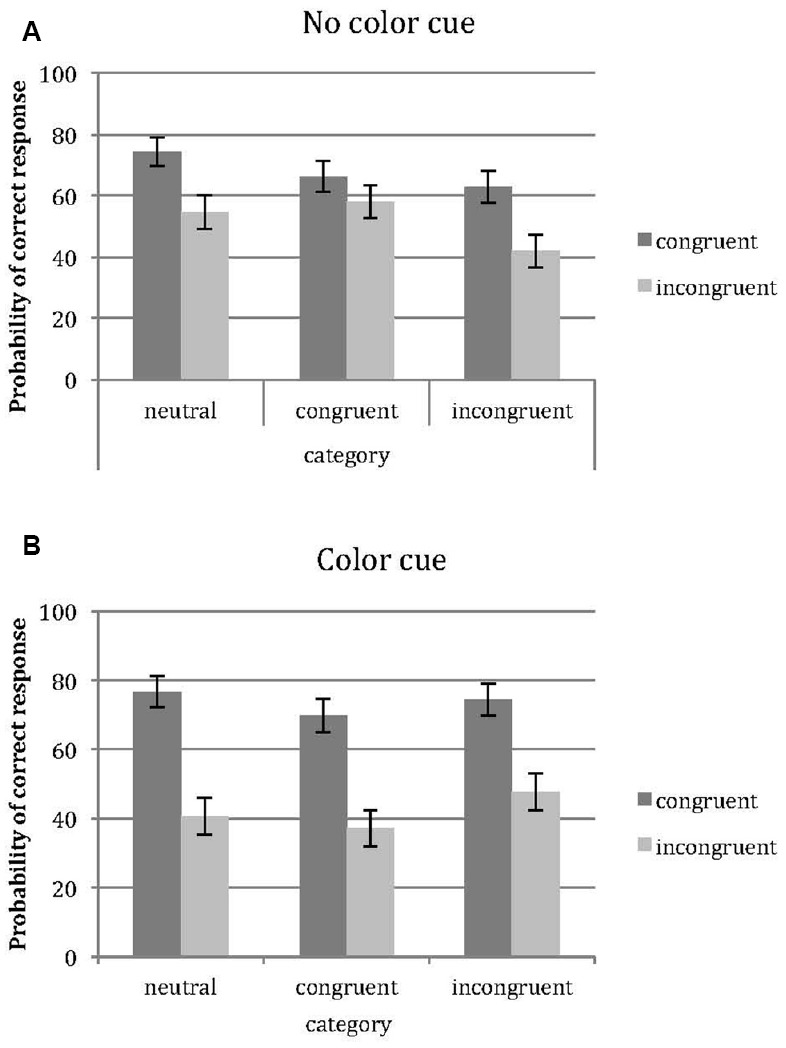
**(A)** Screenshots taken in the environment, from the participant’s perspective. **(B)** Top view of the two different conditions; the color cue condition and the no color cue condition. In both images, the positions of the six sequential objects are indicated. Two different, but highly comparable layouts of objects were used for each participant, evenly divided between the two conditions.

Mean performance scores split up by age group and gender are provided in Table 3. In Figures 2A,B, the overall mean accuracy per condition is provided.

**TABLE 3:** **Mean scores for each task (distance comparison, location memory, order memory) for all participants, divided up by age and gender**.

**Age group (years of age)**	**Distance comparison**	
	**Female**	**Male**
5	41.7 (–)	–
6	50.0 (14)	55.6 (17)
7	45.1 (16)	48.8 (18)
8	54.6 (21)	56.0 (16)
9	55.3 (9)	60.4 (15)
10	55.7 (14)	59.3 (16)
11	67.2 (16)	61.0 (16)
12	63.0 (10)	68.2 (16)
13	43.8 (18)	60.3 (18)
14	63.9 (5)	50 (–)
15	66.7 (12)	70.8 (6)
20	53.1 (25)	67.9 (17)
25	57.8 (15)	63.9 (16)
30	52.8 (32)	60.2 (17)
35	56.7 (20)	53.1 (27)
40	56.9 (16)	54.4 (13)
45	58.3 (19)	61.3 (16)
50	52.6 (17)	59.6 (19)
55	58.3 (8)	73.3 (19)
60	45.8 (6)	–
65	41.7 (–)	50 (0)
70	66.7 (17)	50 (0)
75	58.3 (–)	61.1 (10)

Distance comparison is expressed in percentage accuracy. Standard deviations in parentheses.

First we fit the models and then discuss the parameter estimates. First, the individual main effects of the factors color cue, category, and path were assessed. In comparison to the empty model (step 1) there are significant main effects for path, LR difference test = 176,2, df is 1, *p* < 0.001, and category, LR = 14.6, df is 2, *p* < 0.001, but color cue was not significant, LR = 0.2, df is 1, *p* = 0.65. Next, models including two significant main effects were examined, confirming the two significant main effects of path and category. In comparison to the model with a main effect of path, adding category is significant: LR = 14.6, df = 2, *p* < 0.001. Additional analyses showed no further effects of color cue so in the sequel this variable is ignored. Next, models including a significant two-way interaction were tested. None of these showed significant interactions. Therefore, models including higher order interactions were not considered. The final model at step 2 has two main effects on performance: category and path.

In step 3 age and gender were both added to the model that included main effects for category and path. This model was also significant, LR = 6.8, df is 2, *p* = 0.033, *post hoc* testing revealed a significant effect of gender (*p* < 0.01) and no effect for age group (*p* > 0.10). In step 4 we assessed a random effect for path (Wald test = 616, df is 471, *p* < 0.001), showing that there are individual differences in the path effect. This model was followed up by examining whether gender and age can explain this random effect, but neither of these analyses resulted in significant effects (*p* = 0.436 for gender and *p* = 0.502 for age).

We now turn to the interpretation of the final model, which shows significant effects of path, category, and gender on performance. Performance was higher when path was congruent, compared to incongruent path (marginal effect on performance is a difference of 18.3 %). The effect of category was restricted to the incongruent and neutral condition; performance was significantly higher for the neutral condition (difference is 6.6 %; *p* < 0.001). Performance on the incongruent condition was marginally lower than on the congruent condition (difference is 3.2 %, *p* = 0.052). For the sizes of the effects we refer to Figure 2. Males showed a higher level of accuracy than females (difference is 4.0 %).

## Discussion

In the current study, we examined effects of categorization and path in an interactive, 3D VR setting. In a virtual environment, participants picked up six objects in a circular environment and were asked to compare distances between the objects. A categorization pattern based on the cardinal axes was expected, in particular when these axes were highlighted by color cues along the walls. Furthermore, an effect of path was expected, in which objects that were closer along the path taken between them would be perceived as being metrically closer together.

With regard to categorization, participants were negatively affected when the category was incongruent, compared to when it was neutral or congruent. This pattern was not affected by the presence of color cues. This shows that even without explicit indication, participants employ the cardinal axes of a three dimensional environment, based on their starting point. Moreover, a significant effect of path was also found; objects closer in path were also perceived to be closer metrically. This path congruency effect was not affected by the presence of color cues or category properties of the locations. Notably, performance was at chance level in all incongruent path trial types. This indicates that participants heavily rely on the path taken for their distance comparisons, rather than on the exact object locations.

A very large sample of participants was tested, with ages ranging from 5 to 78, both males and females, in order to study effects of age and gender. The analyses show that effects of both factors are limited. A significant effect of gender illustrated better performance of males compared to females on the distance comparison task. This effect can possibly be explained by the metric nature of this task, as males tend to resort more to Euclidian strategies (see Coluccia and Louse, 2004, for a review).

The effects of categorization and path were not sensitive to age, at least not the range of age of the current sample of participants. However, general ability to correctly compare distances appears to be present between the ages of 8 and 55, as the average performance of participants below and above this age range are at chance level. Clearly, young children (<8 years) have to learn to master the use of cues to effectively orient in space. In turn with older age (>55) this ability declines again.

How do the current findings translate to real life situations? Moving around in three dimensions and memorizing the locations of objects and the relations between them is a common task. The current findings show that the mental representation of the layout of objects is strongly affected by how the environment is explored. When shortest routes are taken, this leads to mostly correct judgments, however, if detours are used, biases in relative distances emerge. Moreover, even in the absence of visible cues to axes of an environment, even a starting point appears to be sufficient to identify cardinal axes in an environment, which consequently affect our representations of object locations. It should be noted that the current results were found in a desktop VR environment, which limits the generalization to actual movement in the real world, as locomotion has been shown to be an influential factor in these processes (e.g., Ruddle and Lessels, 2006).

Taken together, the current study shows that our representations of a three dimensional environment are biased by categorization and the order in which locations are encountered. Even without visual cues to categories we identify the cardinal axes of the environment and experience locations within the same quadrant as being closer together. Furthermore, path congruency has a very strong effect on how distances are perceived. These effects are very similar for participants aged between 5 and 78. Clearly these biases are present from a very early age onward. However, the general ability to estimate distances does show a clear pattern across development and aging.

### Conflict of Interest Statement

The authors declare that the research was conducted in the absence of any commercial or financial relationships that could be construed as a potential conflict of interest.
